# Barriers to Disaster Preparation When Older Adults Are Taking Care of Older Adults

**DOI:** 10.1093/geroni/igaa057.1414

**Published:** 2020-12-16

**Authors:** Zhirui Chen, Zhen Cong

**Affiliations:** The University of Texas at Arlington, Arlington, Texas, United States

## Abstract

This study examined the association between age and barriers for disaster preparation in the U.S., including the moderating effect of caring for an elderly person. Using a sample of 1147 individuals from the 2017 FEMA National Household Survey, we tested age as the key predictor, which had four groups: 18-44 (young age), 45-64 (middle age), 65-74 (young-old), and 75+ (old-old). Binomial logistic regression was conducted to investigate how age and the responsibility to take care of an older adult affected the likelihood of having preparation barriers, controlling for respondents’ gender, education, race, home ownership, disability, and responsibility to take care of an older adult. The results showed that compared with other age groups, young-old were significantly less likely to have barriers for disaster preparation. In addition, taking care of an older adult increased the likelihood of having preparation barriers. Interestingly, relative to the young-old, caring for an older adult presented additional challenges for other age groups to have barriers for preparation. Our findings directed attention to age patterns in barriers for disaster preparation and heterogeneity among older adults, and also highlighted that caring for older adults could exacerbate barriers for disaster preparation. The life course perspective informed the discussion of results, which emphasized on life-span development and linked lives.

